# Supplementary material for: Antidepressant discontinuation before or during pregnancy and risk of psychiatric emergency in Denmark: A population-based propensity score–matched cohort study
Source: PLoS Med. 2022 Jan 31;19(1):e1003895. doi: 10.1371/journal.pmed.1003895 (PMC8843130; doi:10.1371/journal.pmed.1003895)
Supplement: S1 STROBE Checklist — STROBE, Strengthening the Reporting of Observational Studies in Epidemiology. (PDF) [file pmed.1003895.s001.pdf]

S1. STROBE checklist of items that should be included in reports of *cohort studies*

	Item No	Recommendation	Paragraph in manuscript
**Title and abstract**	1	(*a*) Indicate the study's design with a commonly used term in the title or the abstract<br>(*b*) Provide in the abstract an informative and balanced summary of what was done and what was found	In Methods and findings: paragraphs 1-2
**Introduction**			
Background/rationale	2	Explain the scientific background and rationale for the investigation being reported	In Introduction: paragraphs 1-3
Objectives	3	State specific objectives, including any prespecified hypotheses	In Introduction: paragraph 4
**Methods**			
Study design	4	Present key elements of study design early in the paper	In Study population: paragraph 1
Setting	5	Describe the setting, locations, and relevant dates, including periods of recruitment, exposure, follow-up, and data collection	In Study population: paragraph 1, Exposure of interest: paragraphs 1-2, Statistical analysis: paragraph 1
Participants	6	(*a*) Give the eligibility criteria, and the sources and methods of selection of participants. Describe methods of follow-up<br>(*b*) For matched studies, give matching criteria and number of exposed and unexposed	In Study population: paragraph 1, Propensity score-matched cohorts: paragraphs 1-3, Results: paragraphs 1-2
Variables	7	Clearly define all outcomes, exposures, predictors, potential confounders, and effect modifiers. Give diagnostic criteria, if applicable	In Exposure of interest: paragraphs 1-2, Propensity score-matched cohorts: paragraphs 1-3, Outcomes of interest: paragraph 1
Data sources/ measurement	8*	For each variable of interest, give sources of data and details of methods of assessment (measurement). Describe comparability of assessment methods if there is more than one group	In Exposure of interest: paragraphs 1-2, Propensity score-matched cohorts: paragraphs 1-3, Outcomes of interest: paragraph 1, S2 Danish national registers, S3 Definition of suicide attempts, S1-3 Tables
Bias	9	Describe any efforts to address potential sources of bias	In Propensity score-matched cohorts: paragraphs 1-3, Statistical analysis: paragraph 2
Study size	10	Explain how the study size was arrived at	In Study population: paragraph 1, Results: paragraph 2, Figure 1
Quantitative variables	11	Explain how quantitative variables were handled in the analyses. If applicable, describe which groupings were chosen and why	In Propensity score-matched cohorts: paragraphs 1-3
Statistical methods	12	(*a*) Describe all statistical methods, including those used to control for confounding<br>(*b*) Describe any methods used to examine subgroups and interactions<br>(*c*) Explain how missing data were addressed<br>(*d*) If applicable, explain how loss to follow-up was addressed<br>(*e*) Describe any sensitivity analyses	In Statistical analysis: paragraphs 1-2
**Results**			
Participants	13*	(a) Report numbers of individuals at each stage of study—e.g. numbers potentially eligible, examined for eligibility, confirmed eligible, included in the study, completing follow-up, and analysed<br>(b) Give reasons for non-participation at each stage	In Results: paragraphs 1-2, Figure 1

		(c) Consider use of a flow diagram	
Descriptive data	14*	(a) Give characteristics of study participants (e.g. demographic, clinical, social) and information on exposures and potential confounders<br>(b) Indicate number of participants with missing data for each variable of interest<br>(c) Summarise follow-up time (e.g., average and total amount)	In Results: paragraph 1, Table 1-2, S4 Table
Outcome data	15*	Report numbers of outcome events or summary measures over time	In results: paragraphs 3-4, Table 2
Main results	16	(*a*) Give unadjusted estimates and, if applicable, confounder-adjusted estimates and their precision (e.g., 95% confidence interval). Make clear which confounders were adjusted for and why they were included<br>(*b*) Report category boundaries when continuous variables were categorized<br>(*c*) If relevant, consider translating estimates of relative risk into absolute risk for a meaningful time period	In results: paragraphs 3-4, Figure 2, Table 2
Other analyses	17	Report other analyses done—e.g. analyses of subgroups and interactions, and sensitivity analyses	In results: paragraph 5, S5-6 Tables
**Discussion**			
Key results	18	Summarise key results with reference to study objectives	In Discussion: paragraph 1
Limitations	19	Discuss limitations of the study, taking into account sources of potential bias or imprecision. Discuss both direction and magnitude of any potential bias	In Discussion: paragraph 8
Interpretation	20	Give a cautious overall interpretation of results considering objectives, limitations, multiplicity of analyses, results from similar studies, and other relevant evidence	In Discussion: paragraphs 1–8
Generalisability	21	Discuss the generalisability (external validity) of the study results	In Discussion: paragraphs 7-8
Funding	22	Give the source of funding and the role of the funders for the present study and, if applicable, for the original study on which the present article is based	In Acknowledgement and Funding

*Give information separately for exposed and unexposed groups.

Note: An Explanation and Elaboration article discusses each checklist item and gives methodological background and published examples of transparent reporting. The STROBE checklist is best used in conjunction with this article (freely available on the Web sites of PLoS Medicine at http://www.plosmedicine.org/, Annals of Internal Medicine at http://www.annals.org/, and Epidemiology at http://www.epidem.com/). Information on the STROBE Initiative is available at http://www.strobe-statement.org.
