# Supplementary material for: Antidepressant discontinuation before or during pregnancy and risk of psychiatric emergency in Denmark: A population-based propensity score–matched cohort study
Source: PLoS Med. 2022 Jan 31;19(1):e1003895. doi: 10.1371/journal.pmed.1003895 (PMC8843130; doi:10.1371/journal.pmed.1003895)
Supplement: S1 Text — (PDF) [file pmed.1003895.s002.pdf]

## S1. Detailed description of Danish national registers used in this study

**The Danish Civil Registration System** [1] was established for administrative use on April 2, 1968. All residents alive and living in Denmark were registered. This system includes a unique 10-digit individual personal identification number (i.e., CPR number), gender, date of birth, marital status, spouse, parents, children, and vital statistics. The information is of very high quality [1]. This unique CPR number can be used for linkage between all national registers.

**The Danish Medical Birth Registry** [2] was established in 1968 and has been computerized since 1973. The registry comprises data on all live births and stillbirths born to mothers residing in Denmark. Since 1978, information on gestational age and birth weight for children born in Denmark has been included. Gestational age was recorded in complete weeks until 1996 and in days afterward. Birth weight was registered in categories of 250 grams in 1978, in categories of 10 grams from 1979, and in exact grams after 1990. Data on age and personal identification of mother and child, gestational age, and birth weight are of good quality.

**The Danish National Patient Register** [3] contains information on visits to all public hospitals. The population has relatively equal access to services. The register includes information on inpatients since 1977, and since 1995 outpatients and emergency room visits are included. Data on psychiatric hospital contact are added as well.

**The Danish Psychiatric Central Research Register** [4] was established as an electronic database in 1969. The register holds information on all treatments at psychiatric hospitals and psychiatric wards in general hospitals since 1969. In 1995, data on emergency room visits and

outpatient treatments were included. From 1977–1993, the International Classification of Diseases, 8th revision (ICD-8) was used and 10th revision (ICD-10) from 1994 onwards for the Danish Psychiatric Central Research Register and the Danish National Patient Register.

**The Danish National Prescription Registry** [5] was established in 1995 and contains information on all prescriptions dispensed at community pharmacies in Denmark. The register includes data on drug class (Anatomical Therapeutic Chemical [ATC] classification system), the dispensing date, defined daily doses per packet, and packets dispensed.

## References


1. Pedersen CB. The Danish Civil Registration System. *Scand J Public Health*. 2011;39(7 Suppl):22-5. Epub 2011/08/04. doi: 10.1177/1403494810387965. PMID: 21775345.
2. Knudsen LB, Olsen J. The Danish Medical Birth Registry. *Dan Med Bull.* 1998;45(3):320-3. Epub 1998/07/24. PMID: 9675544.
3. Andersen TF, Madsen M, Jorgensen J, Mellemkjoer L, Olsen JH. The Danish National Hospital Register. A valuable source of data for modern health sciences. *Dan Med Bull*. 1999;46(3):263-8. Epub 1999/07/28. PMID: 10421985.
4. Mors O, Perto GP, Mortensen PB. The Danish Psychiatric Central Research Register. *Scand J Public Health*. 2011;39(7 Suppl):54-7. Epub 2011/08/04. doi: 10.1177/1403494810395825. PMID: 21775352.
5. Kildemoes HW, Sorensen HT, Hallas J. The Danish National Prescription Registry. *Scand J Public Health*. 2011;39(7 Suppl):38-41. Epub 2011/08/04. doi: 10.1177/1403494810394717. PMID: 21775349.
