# Supplementary material for: Antidepressant discontinuation before or during pregnancy and risk of psychiatric emergency in Denmark: A population-based propensity score–matched cohort study
Source: PLoS Med. 2022 Jan 31;19(1):e1003895. doi: 10.1371/journal.pmed.1003895 (PMC8843130; doi:10.1371/journal.pmed.1003895)
Supplement: S2 Text — (PDF) [file pmed.1003895.s003.pdf]

S2. Charlson comorbidity index

(1) Comorbidity category assigned a score of 1: Myocardial infarction, congestive heart failure, peripheral vascular disease, cerebrovascular disease, dementia, chronic pulmonary disease, connective tissue disease, ulcer disease, mild liver disease, type 1 and type 2 diabetes

(2) Comorbidity category assigned a score of 2: Hemiplegia, moderate to severe renal disease, diabetes with end-organ damage type 1 and type 2, any tumor, leukemia, and lymphoma

(3) Comorbidity category assigned a score of 3: Moderate to severe liver disease

(4) Comorbidity category assigned a score of 6: Metastatic solid tumor and AIDs
