# Supplementary material for: Antidepressant discontinuation before or during pregnancy and risk of psychiatric emergency in Denmark: A population-based propensity score–matched cohort study
Source: PLoS Med. 2022 Jan 31;19(1):e1003895. doi: 10.1371/journal.pmed.1003895 (PMC8843130; doi:10.1371/journal.pmed.1003895)
Supplement: S3 Text — (PDF) [file pmed.1003895.s004.pdf]

S3. Definition of suicide attempts

Suicide attempts before antidepressant discontinuation were defined as inpatient or outpatient contact of suicide attempts identified from the Danish Psychiatric Central Research Register and the Danish National Patient Register [1]. Individuals were considered to have made a suicide attempt if they fulfilled one of the following criteria:

(1) The main diagnosis of suicide attempt or deliberate self-harm (ICD-8 code: E950–E959; ICD-10: X60–84)

(2) The main diagnosis of poisoning with weak analgesics, epileptic drugs, or carbon monoxide (ICD-10: T39, T42, T43, and T58)

(3) Hospital contacts where the primary diagnosis is a psychiatric disorder (any from the ICD-10 F chapter and 290–315 in ICD-8) and the secondary diagnosis is intoxication (ICD-10: T36–T50 and T52–T60) or lesions at the forearm, wrist, or hand (ICD-10: S51, S55, S59, S61, S65, and S69)

Reference

1. Gasse C, Danielsen AA, Pedersen MG, Pedersen CB, Mors O, Christensen J. Positive predictive value of a register-based algorithm using the Danish National Registries to identify suicidal events. *Pharmacoepidemiol Drug Saf.* 2018;27(10):1131-1138.
