# Supplementary material for: Antidepressant discontinuation before or during pregnancy and risk of psychiatric emergency in Denmark: A population-based propensity score–matched cohort study
Source: PLoS Med. 2022 Jan 31;19(1):e1003895. doi: 10.1371/journal.pmed.1003895 (PMC8843130; doi:10.1371/journal.pmed.1003895)
Supplement: S1 Table — (PDF) [file pmed.1003895.s005.pdf]

S1 Table. The ICD-8 or ICD-10 codes for subgroup diagnosis of psychiatric comorbidities.

Name of disorders or medications	ICD-8 codes	ICD-10 codes
Any psychiatric disorders	290–309	F00–F99 excluding F70–F79
Substance abuse disorder	291.X9, 294.39, 303.X9, 303.20, 303.28, 303.90, and 304.X9	F10–F19
Schizophrenia and related disorders, abbreviated hereafter as schizophrenia	295.X9, 296.89, 297.X9, 298.29–298.99, 299.04, 299.05, 299.09, and 301.83	F20–F29
Bipolar disorder	296.19, 296.39, and 298.19	F30–31
Depression	296.09, 296.29, 298.09, and 300.49	F32–33
Other mood disorders except for bipolar and unipolar disorders;	296.X9 and 301.19 excluding 296.09, 296.19, 296.29, and 296.39	F34–39
Neurotic, stress-related, and somatoform disorders	300.X9, 305.X9, 305.68, and 307.99 excluding 300.49	F40–F48
Personality disorders	300.19, 301.49, 301.59, 301.69, 301.79, 301.80, 301.81, 301.82, 301.83, and 301.84	F60–F69
Behavioral and emotional disorders with onset usually occurring in childhood and adolescence	306.X9, and 308.0X	F90–F98
