# Supplementary material for: Antidepressant discontinuation before or during pregnancy and risk of psychiatric emergency in Denmark: A population-based propensity score–matched cohort study
Source: PLoS Med. 2022 Jan 31;19(1):e1003895. doi: 10.1371/journal.pmed.1003895 (PMC8843130; doi:10.1371/journal.pmed.1003895)
Supplement: S2 Table — (PDF) [file pmed.1003895.s006.pdf]

## S2 Table. Classes of antidepressant treatment during pregnancy.

| **Antidepressants** | **ATC code** |
|---|---|
| **Selective serotonin reuptake inhibitors** | N06AB |
| Citalopram | N06AB04 |
| Sertraline | N06AB06 |
| Fluoxetine | N06AB03 |
| Paroxetine | N06AB05 |
| Escitalopram | N06AB10 |
| Fluvoxamine | N06AB08 |
| More than one SSRI | - |
| **Serotonin-norepinephrine reuptake inhibitors** | N06AX |
| **Tricyclic antidepressants and others** (e.g., monoamine oxidase inhibitors and tetracyclic antidepressants) | N06AA, N06AG, and N06AF |
