# Supplementary material for: Antidepressant discontinuation before or during pregnancy and risk of psychiatric emergency in Denmark: A population-based propensity score–matched cohort study
Source: PLoS Med. 2022 Jan 31;19(1):e1003895. doi: 10.1371/journal.pmed.1003895 (PMC8843130; doi:10.1371/journal.pmed.1003895)
Supplement: S3 Table — ATC, Anatomical Therapeutic Chemical. (PDF) [file pmed.1003895.s007.pdf]

S3 Table. Anatomical therapeutic chemical (ATC) codes for co-medications in the 90 days prior to pregnancy.

Name of disorders or medications	ATC codes
Opioid	N02A
Antiepileptics	N03
Antipsychotics	N05A
Benzodiazepine	N05BA, N05CD and N05CF
Anxiolytics excluding benzodiazepine	N05B excluding N05BA
Barbiturates	N05CA and N05CB
Other hypnotics	N05C excluding N05CB, N05CD, and N05CF
