# Supplementary material for: Antidepressant discontinuation before or during pregnancy and risk of psychiatric emergency in Denmark: A population-based propensity score–matched cohort study
Source: PLoS Med. 2022 Jan 31;19(1):e1003895. doi: 10.1371/journal.pmed.1003895 (PMC8843130; doi:10.1371/journal.pmed.1003895)
Supplement: S4 Table — (PDF) [file pmed.1003895.s008.pdf]

S4 Table. Characteristics of the study population before propensity score matching. Values are numbers (%) unless stated otherwise.

Characteristics	Discontinuation before pregnancy (N=4,368)	Discontinuation during pregnancy (N=13,258)	Continuation group (N=5,563)	Absolute standardized differences between discontinuation before pregnancy and continuation	Absolute standardized differences between discontinuation during pregnancy and continuation	Absolute standardized differences between discontinuation before pregnancy and during pregnancy
**Age at the index pregnancy**						
18–25	1,226 (28.1)	3,623 (27.3)	1,178 (21.2)	0.17	0.15	0.02
26–29	1,133 (25.9)	3,405 (25.7)	1,568 (28.2)			
30–34	1,249 (28.6)	3,923 (29.6)	1,845 (33.2)			
≥35	760 (17.4)	2,307 (17.4)	972 (17.5)			
**Parity**						
1st	1,962 (44.9)	6,125 (46.2)	2,727 (49.0)	0.08	0.06	0.03
2nd and above	2,406 (55.1)	7,133 (53.8)	2,836 (51.0)			
**Marital status**						
Married or cohabiting	2,960 (67.8)	8,792 (66.3)	3,970 (71.4)	0.08	0.11	0.03
Single, divorced or widowed	1,408 (32.2)	4,466 (33.7)	1,593 (28.6)			
**Level of education**						
Mandatory school comprising 9 school years	1,623 (37.2)	4,637 (35.0)	1,408 (25.3)	0.27	0.23	0.05
Above mandatory school	2,629 (60.2)	8,208 (61.9)	4,046 (72.7)			
Unknown	116 (2.7)	413 (3.1)	109 (2.0)			
**Age at first affective episode**						
≤18	419 (9.6)	1,540 (11.6)	784 (14.1)	0.29	0.23	0.09
19–25	1,742 (39.9)	5,536 (41.8)	2,626 (47.2)			
26–29	1,079 (24.7)	2,944 (22.2)	1,255 (22.6)			
30–34	836 (19.1)	2,431 (18.3)	735 (13.2)			
≥35	292 (6.7)	807 (6.1)	163 (2.9)			
**History of suicide attempts before pregnancy**	317 (7.3)	1,061 (8.0)	483 (8.7)	0.05	0.02	0.03
**Psychiatric diagnosis before pregnancy**						
Substance abuse disorder	133 (3.0)	478 (3.6)	220 (4.0)	0.05	0.02	0.03
Schizophrenia	21 (0.5)	68 (0.5)	33 (0.6)	0.02	0.01	0.00
Bipolar disorder	26 (0.6)	140 (1.1)	113 (2.0)	0.13	0.08	0.05
Depression	604 (13.8)	2,260 (17.0)	1,506 (27.1)	0.33	0.24	0.09

Other mood disorder	35 (0.8)	170 (1.3)	107 (1.9)	0.10	0.05	0.05
Neurotic, stress-related and somatoform disorders	838 (19.2)	3,107 (23.4)	1,760 (31.6)	0.29	0.18	0.10
Personality disorders	490 (11.2)	1,692 (12.8)	986 (17.7)	0.19	0.14	0.05
Child onset psychiatric disorders	99 (2.3)	370 (2.8)	156 (2.8)	0.03	0.00	0.03
Other disorders	348 (8.0)	1,242 (9.4)	769 (13.8)	0.19	0.14	0.05
**Non-psychiatric comorbidity scores**						
0	3,867 (88.5)	11,613 (87.6)	4,848 (87.1)	0.04	0.02	0.03
1 point	396 (9.1)	1,301 (9.8)	572 (10.3)			
≥2 point	105 (2.4)	344 (2.6)	143 (2.6)			
**Number of previous psychiatric emergencies prior to 90 days before pregnancy**						
0	3,627 (83.0)	10,602 (80.0)	3,971 (71.4)	0.29	0.21	0.09
1	385 (8.8)	1,333 (10.1)	693 (12.5)			
2	146 (3.3)	561 (4.2)	347 (6.2)			
3-4	123 (2.8)	402 (3.0)	293 (5.3)			
≥5	87 (2.0)	360 (2.7)	259 (4.7)			
**Psychiatric emergency in the 90 days before pregnancy**	85 (1.9)	311 (2.3)	114 (2.0)	0.01	0.02	0.03
**Classes of antidepressant treatment in the 90 days before pregnancy** a						
SSRIs	2,526 (57.8)	10,542 (79.5)	4,663 (83.8)	0.60	0.11	0.48
SNRIs	1,520 (34.8)	2,692 (20.3)	960 (17.3)	0.41	0.08	0.33
TCAs or MAIOs	677 (15.5)	609 (4.6)	193 (3.5)	0.42	0.06	0.37
**Co-medications in the 90 days before pregnancy**						
Benzodiazepine	396 (9.1)	1,242 (9.4)	560 (10.1)	0.03	0.02	0.01
Anxiolytics excluding benzodiazepine	9 (0.2)	32 (0.2)	14 (0.3)	0.01	0.00	0.01
Antipsychotics	194 (4.4)	766 (5.8)	471 (8.5)	0.16	0.10	0.06
Opioid	295 (6.8)	573 (4.3)	213 (3.8)	0.13	0.02	0.11
Antiepileptics	150 (3.4)	427 (3.2)	316 (5.7)	0.11	0.12	0.01
Other hypnotics	19 (0.4)	56 (0.4)	20 (0.4)	0.01	0.01	0.00
**Calendar year at the index pregnancy**						
1996–2004	1,573 (36.0)	3,725 (28.1)	1,056 (19.0)	0.39	0.25	0.21
2005–2009	1,770 (40.5)	5,373 (40.5)	2,846 (51.2)			
2011–2015	1,025 (23.5)	4,160 (31.4)	1,661 (29.9)			

[a] The number of classes of antidepressant does not add up to the total number of women since some women received more than one class of antidepressants.
Abbreviations: SSRIs, selective serotonin reuptake inhibitor; SNRIs, serotonin-norepinephrine reuptake inhibitors; TCAs, tricyclic antidepressants; MAOIs, monoamine oxidase inhibitors.
