# Supplementary material for: Antidepressant discontinuation before or during pregnancy and risk of psychiatric emergency in Denmark: A population-based propensity score–matched cohort study
Source: PLoS Med. 2022 Jan 31;19(1):e1003895. doi: 10.1371/journal.pmed.1003895 (PMC8843130; doi:10.1371/journal.pmed.1003895)
Supplement: S5 Table — (PDF) [file pmed.1003895.s009.pdf]

**S5 Table. Risk of psychiatric emergency associated with antidepressant discontinuation in propensity score matched cohort analyses using a less restrictive 30 days grace period.**[a]

| Matched groups according to time of discontinuation of the exposed group | Antidepressant discontinuation group | | | | Antidepressant continuation group | | | | Unadjusted hazard ratios (95% CI) | Adjusted hazard ratios (95%CI) [b] | P-values for adjusted analyses |
|---|---|---|---|---|---|---|---|---|---|---|---|
| | No of women | No of events | Person-years | Incidence/1000 person-years | No of women | No of events | Person-years | Incidence/1000 person-years | | | |
| **Antidepressant discontinuation before pregnancy** | 1,762 | 44 | 2,194.15 | 20.1 | 1,762 | 63 | 2,146.80 | 29.3 | 0.69 (0.47–1.02) | 0.66 (0.44–0.99) | 0.044 |
| During pregnancy | 1,762 | 20 | 1327.80 | 15.1 | 1,762 | 38 | 1,290.55 | 29.4 | 0.53 (0.31–0.90) | 0.41 (0.22–0.79) | 0.008 |
| Within 6 months postpartum | 1,704 | 23 | 847.78 | 27.1 | 1,704 | 24 | 846.43 | 28.4 | 0.96 (0.53–1.72) | 1.00 (0.55–1.80) | 0.994 |
| **Antidepressant discontinuation during pregnancy** | 6,446 | 215 | 6,165.84 | 34.9 | 6,446 | 186 | 6,113.73 | 30.4 | 1.18 (0.95–1.42) | 1.16 (0.96–1.41) | 0.156 |
| During pregnancy | 6,446 | 91 | 3,021.08 | 30.1 | 6,446 | 73 | 2,954.95 | 24.7 | 1.22 (0.92–1.65) | 1.26 (0.93–1.72) | 0.138 |
| Within 6 months postpartum | 6,281 | 121 | 3,108.97 | 38.9 | 6,281 | 110 | 3,115.82 | 35.3 | 1.09 (0.83–1.41) | 1.08 (0.82–1.42) | 0.576 |

[a] The numbers of during pregnancy and within 6 months postpartum do not add up to the whole period since only matched individuals contribute to the analyses; [b] adjustment for imbalanced variables: level of education status and the use of TCAs or MAOIs in the 90 days before pregnancy for the estimate of antidepressant discontinuation before pregnancy; the level of education and age at first affective disorder for the estimate of antidepressant discontinuation during pregnancy.
