# Supplementary material for: Antidepressant discontinuation before or during pregnancy and risk of psychiatric emergency in Denmark: A population-based propensity score–matched cohort study
Source: PLoS Med. 2022 Jan 31;19(1):e1003895. doi: 10.1371/journal.pmed.1003895 (PMC8843130; doi:10.1371/journal.pmed.1003895)
Supplement: S6 Table — (PDF) [file pmed.1003895.s010.pdf]

S6 Table. Risk of psychiatric emergency associated with antidepressant discontinuation in propensity score-matched cohort analyses including age at pregnancy and age at first affective disorders as linear splines.

Matched groups according to time of discontinuation of the exposed group	Antidepressant discontinuation group				Antidepressant continuation group				Unadjusted hazard ratios (95% CI)	Adjusted hazard ratios (95%CI) [b]	P-values for the adjusted analyses
	No of women	No of events	Person-years	Incidence /1000 person-years	No of women	No of events	Person-years	Incidence /1000 person-years			
**Antidepressant discontinuation before pregnancy**	2,635	75	3,268.74	22.9	2,635	89	3,221.03	27.6	0.83 (0.61–1.14)	0.81 (0.58–1.14)	0.231
During pregnancy	2,635	38	1,978.26	19.2	2,635	48	1,936.12	24.8	0.79 (0.52–1.21)	0.78 (0.50–1.22)	0.269
Within 6 months postpartum	2,549	35	1,266.97	27.6	2,549	40	1,265.97	31.6	0.91 (0.55–1.50)	0.93 (0.53–1.61)	0.789
**Antidepressant discontinuation during pregnancy**	5,438	194	5,313.85	36.5	5,438	158	5,279.72	29.9	1.28 (1.03–1.58)	1.27 (1.01–1.59)	0.040
During pregnancy	5,438	82	2,665.30	30.8	5,438	61	2,615.64	23.3	1.40 (1.00–1.96)	1.48 (1.03–2.12)	0.033
Within 6 months postpartum	5,295	109	2,619.56	41.6	5,295	96	2,624.95	36.6	1.24 (0.92–1.68)	1.18 (0.85–1.64)	0.316

[a] The numbers of during pregnancy and within 6 months postpartum do not add up to the whole period since only matched individuals contribute to the analyses;
[b] adjustment for imbalanced variables: level of education status and the use of TCAs or MAOIs in the 90 days before pregnancy for the estimate of antidepressant discontinuation before pregnancy; the level of education and age at first affective disorder for the estimate of antidepressant discontinuation during pregnancy.
