# Supplementary figures and images for: Antidepressant discontinuation before or during pregnancy and risk of psychiatric emergency in Denmark: A population-based propensity score–matched cohort study
Source: PLoS Med. 2022 Jan 31;19(1):e1003895. doi: 10.1371/journal.pmed.1003895 (PMC8843130; doi:10.1371/journal.pmed.1003895)

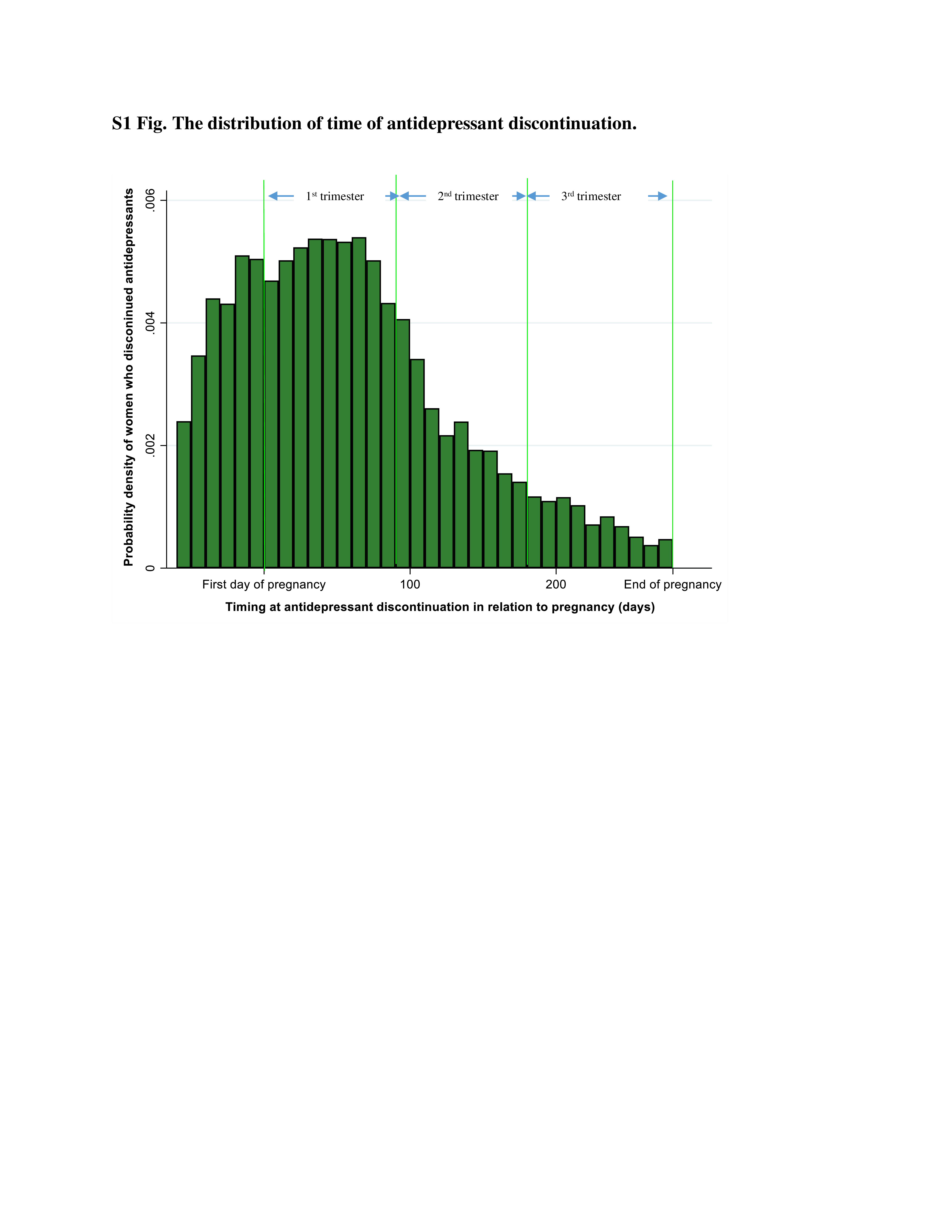

Supplement: S1 Fig — (TIFF) [file pmed.1003895.s011.tiff]

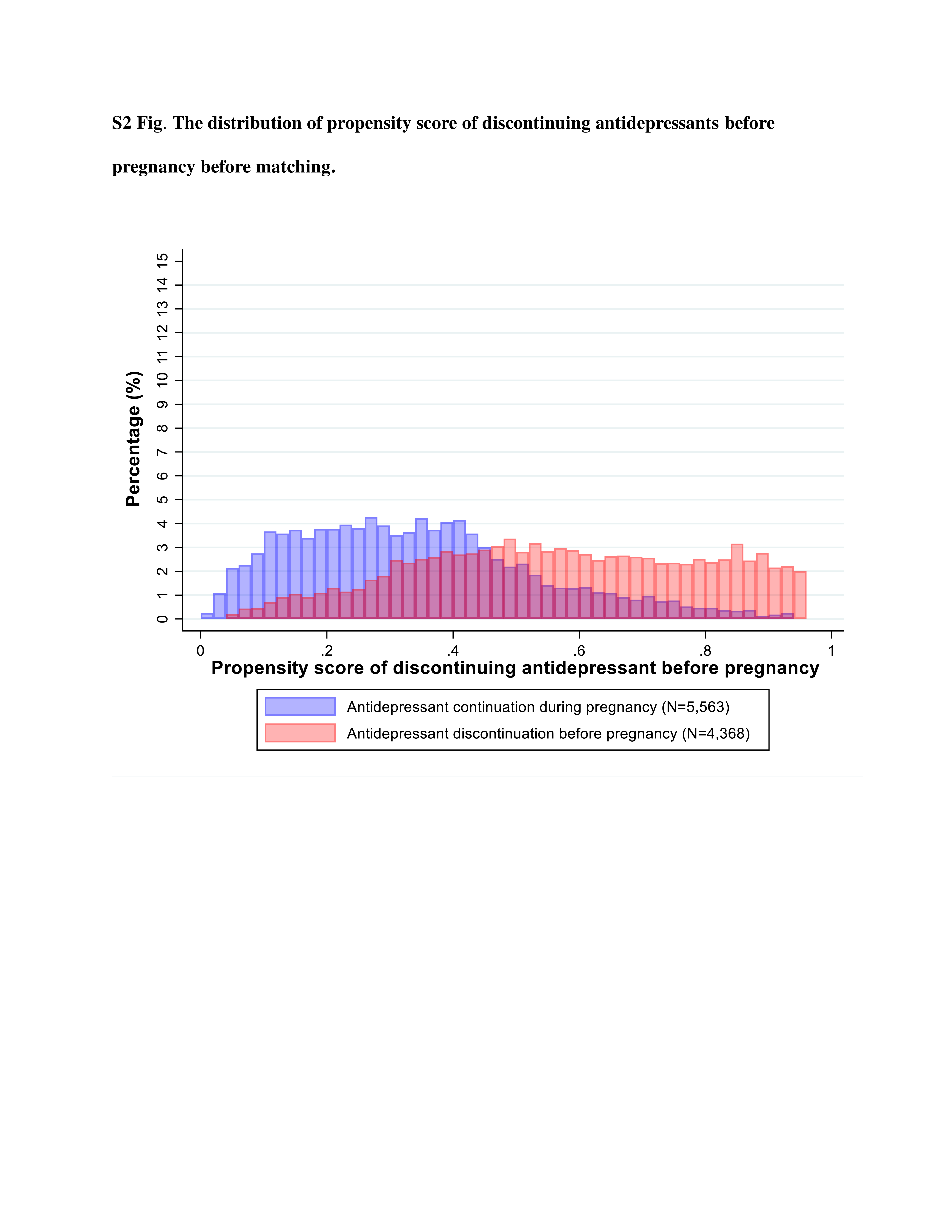

Supplement: S2 Fig — (TIFF) [file pmed.1003895.s012.tiff]

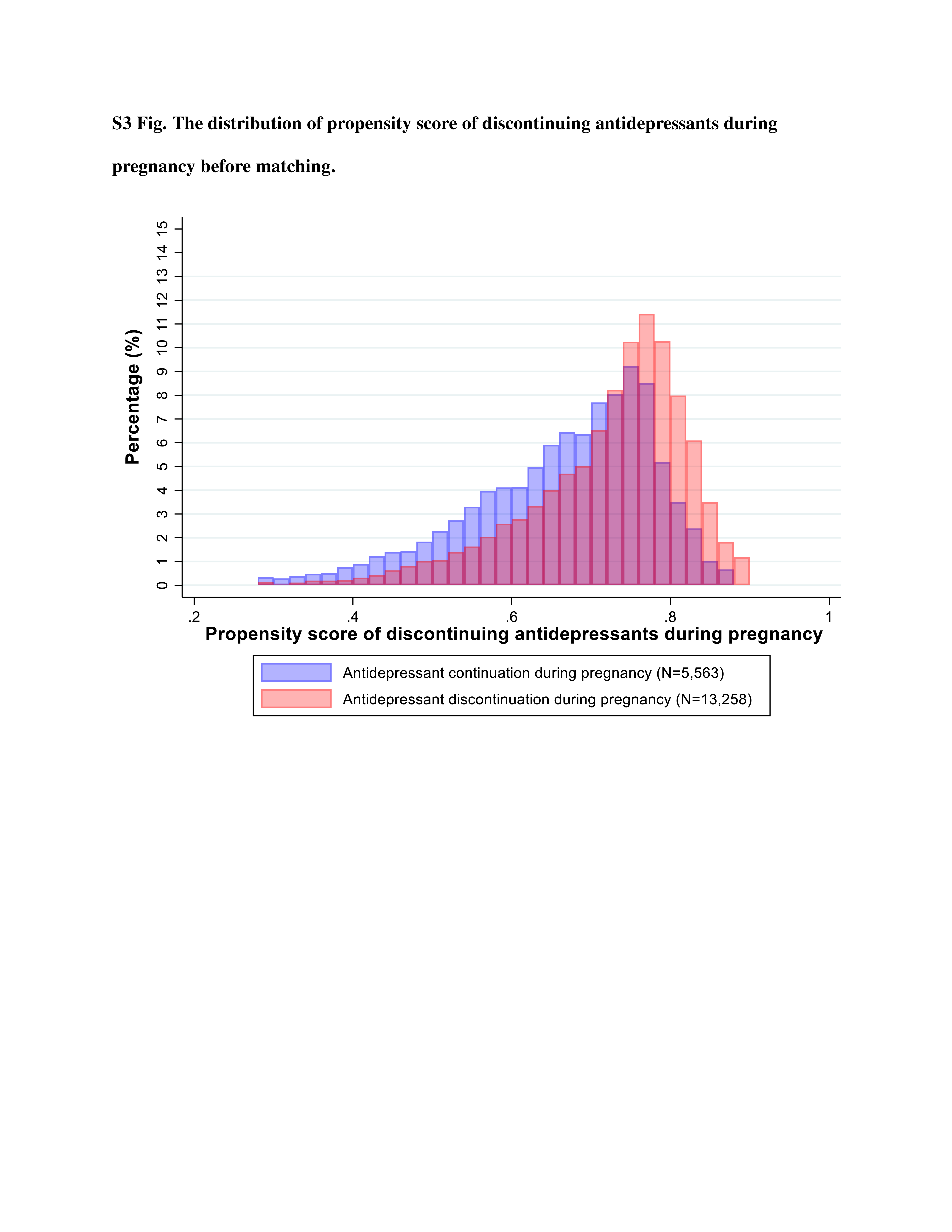

Supplement: S3 Fig — (TIFF) [file pmed.1003895.s013.tiff]
