# Supplementary material for: Antidepressant discontinuation before or during pregnancy and risk of psychiatric emergency in Denmark: A population-based propensity score–matched cohort study
Source: PLoS Med. 2022 Jan 31;19(1):e1003895. doi: 10.1371/journal.pmed.1003895 (PMC8843130; doi:10.1371/journal.pmed.1003895)
Supplement: Attachment — Submitted filename: rebuttal letter.docx [file pmed.1003895.s014.docx]

Dear editors,

Thank you for sending us the comments and feedback from the editors and reviewers. We would like to thank everyone for taking the time to read our manuscript and for providing us with valuable and constructive input. We appreciate the opportunity to revise and resubmit our manuscript, which we believe is improved based on the suggested revisions. Please find below a detailed point-by-point response to all comments (editors and reviewers' comments in black, our replies in blue). The line numbers mentioned are based on the revised manuscript with track changes.

**Requests from the editors:**

1. We note that there are major concerns raised by the reviewers, especially reviewer 3, relating to the validity of the analyses. Please do respond to and address the comments of all the reviewers.

**Response**: We have included a point-by-point reply to address the concerns raised by the reviewers.

2. Data availability statement: The Data Availability Statement (DAS) requires revision. For each data source used in your study:

a) If the data are freely or publicly available, note this and state the location of the data: within the paper, in Supporting Information files, or in a public repository (include the DOI or accession number).

b) If the data are owned by a third party but freely available upon request, please note this and state the owner of the data set and contact information for data requests (web or email address). Note that a study author cannot be the contact person for the data.

c) If the data are not freely available, please describe briefly the ethical, legal, or contractual restriction that prevents you from sharing it. Please also include an appropriate contact (web or email address) for inquiries (again, this cannot be a study author).

**Response**: We have now expanded the data availability statement in our previous version to include an appropriate contact person. It now reads:

"*The study was based on Danish nationwide registers, and the data are not collected for a specific research project. According to the Danish legislation, individual-level data can be accessed only through secure servers**, Denmark Statistics, where download or export of individual-level Information is prohibited. Only aggregated data can be shared to ensure complete anonymity and protection of individuals included in the studies. For any inquiries, please contact Professor Carsten Bøcker Pedersen (cbp@econ.au.dk) at the National Centre for Register-based Research, Aarhus University, Denmark.*"

3. Throughout: Please include line numbers with the revised version.

**Response:** We have included line numbers in the revised version.

4. Abstract: Please combine the Methods and Findings sections into one section, "Methods and findings".

**Response**: We have combined the Methods and Findings sections as suggested.

5. Abstract: Methods and Findings: Please include the population and setting, number of participants, and years during which the study took place.

**Response**: We have now included this information in the abstract as suggested.

6. Abstract: Methods and Findings: Please include the important independent variables that are adjusted for in the analyses.

**Response**: We have included information on the independent variables to generate the propensity score in the abstract.

7. Abstract: Methods and Findings: Please quantify the main results with both 95% CIs and p values.

**Response**: We have added p-values to the abstract as suggested.

8. Abstract: Methods and Findings: In the last sentence of the Abstract Methods and Findings section, please describe the main limitation(s) of the study's methodology.

**Response**: We have added one sentence in the abstract to describe the main limitations of the study. It reads, "*Study limitations include lack of information on indications for antidepressant treatment and reasons for discontinuation."*

9. Throughout: Please use square brackets for in-text citations, placed before the sentence punctuation. Where multiple references are included within brackets, please do not include spaces.

**Response**: We have formatted the references as requested.

10. Methods: Please ensure that the study is reported according to the STROBE guideline, and include the completed STROBE checklist as Supporting Information. Please add the following statement, or similar, to the Methods: "This study is reported as per the Strengthening the Reporting of Observational Studies in Epidemiology (STROBE) guideline (S1 Checklist)."

The STROBE guideline can be found here: http://www.equator-network.org/reporting-guidelines/strobe/

When completing the checklist, please use section and paragraph numbers, rather than page numbers.

**Response**: We have added the statement in the Methods section (Methods section, Line 104–105), and included the STROBE guideline in the supplementary materials (S1).

11. Methods: Did your study have a prospective protocol or analysis plan? Please state this (either way) early in the Methods section.

a) If a prospective analysis plan (from your funding proposal, IRB or other ethics committee submission, study protocol, or other planning document written before analyzing the data) was used in designing the study, please include the relevant prospectively written document with your revised manuscript as a Supporting Information file to be published alongside your study, and cite it in the Methods section. A legend for this file should be included at the end of your manuscript.

b) If no such document exists, please make sure that the Methods section transparently describes when analyses were planned, and when/why any data-driven changes to analyses took place.

c) In either case, changes in the analysis-- including those made in response to peer review comments-- should be identified as such in the Methods section of the paper, with rationale.

**Response**: For the present study, we planned our analyses on a detailed discussion among the authors and agreed on an outline for how the work would be done, which leads to the methods section included in the manuscript. We have described our methods in detail to ensure transparency and replicability and have also justified the changes in the analysis.

12. Methods: Please provide more detail on the Danish nationwide registers used for the cohort study.

**Response**: We have added a detailed description of the Danish nationwide registers in the supplementary material (S2).

13. Methods: Please comment on whether it is possible to examine recurrent illness that may not necessarily present in the ER/ hospital perhaps due to being less severe relative to those illnesses that may present in the ER/hospital.

**Response**: Our study focuses on severe exacerbation of symptoms/mental health crisis, i.e., psychiatric emergency or inpatient treatment, for which there is no risk of misclassification. Clinically, our chosen outcome of interest (emergency/inpatient treatment in pregnant women and new mothers) is highly relevant, given this is an extremely worrying situation for a group of vulnerable women (pregnant or new mothers), who are at an increased risk of suicide and self-harm [1, 2].

We fully acknowledge that it would be interesting in future studies with distinct designs to explore less severe outcomes after antidepressant discontinuation. We have included these considerations in our discussion section (Line 325–329). It reads,

*" Furthermore, our results should be interpreted with caution because psychiatric emergency is a severe outcome. In contrast, a larger proportion of these women may have a recurrent episode that does not necessarily lead to emergency room visits or admissions. It would be interesting to investigate less severe outcomes such as worsening of symptoms or affective instability in future prospective clinical studies."*

14. Results: Please present both 95% CIs and p values for all results described in the text; for example, for the association of antidepressant discontinuation with psychiatric emergency risk.

**Response**: We have included both the 95% CIs and p values for all results described in the text, as suggested.

15. Results: Please note where the results to support this statement are presented: "The risk of psychiatric emergency associated with antidepressant discontinuation before pregnancy did not differ between the pregnancy and the postpartum periods" (please note where the results for the interaction test can be found).

**Response**: We apologize for the misunderstanding. We described the results for the interaction test in the text but not in Table 2. We have rephrased the sentences that detail these results for clarity (Line 262–266).

16. Results: Please clarify for the analysis of those who discontinued medication during pregnancy, that the interaction between time of observing the outcome of psychiatric emergency (during pregnancy or postpartum) did not reach significance. This seems to suggest that the different risk noted should be interpreted with caution given the lack of an interaction.

**Response**: We agree with the editor that the interaction was not statistically significant, and the conclusion regarding the interaction should be toned down. Therefore, we deleted the sentence on no increased risk in the postpartum period from the first paragraph in the discussion section. Moreover, we have included a paragraph to explain the different risks observed in pregnancy and in the postpartum period as suggested by Reviewer 3 but interpreted the results with caution as the editor suggested (Line 317–323).

17. Results: "When applying a less restrictive grace period of 30-days, in contrast to that of 14-days applied in the main analyses, findings were similar; however, associations were attenuated (HR=1.16, 95%CI:0.96–1.41)..." Please clarify which associations are being referred to here.

**Response**: We have rephrased the sentence for clarification (Line 269–274).

18. Results: Sensitivity analysis: Please include in the discussion some interpretation on why the association between discontinuation during pregnancy and psychiatric emergency might reverse direction with the 30 day rather than 14 day grace period. Please note in the text the sensitivity analyses for which associations observed in main analyses were attenuated/statistical significance no longer apparent.

**Response**: We defined antidepressant discontinuation using two different grace periods, 14 days [3] and 30 days [4], according to the definitions suggested by two world-leading pharmacoepidemiological groups in the USA. We have rephrased the sentence for clarification on which association was attenuated and no longer statistically significant in the text (Line 269–274).

In the primary analysis, when defining antidepressant discontinuation using 14 days' grace period, we found an HR of 0.84 (95% CI: 0.61–1.16) between antidepressant discontinuation before pregnancy and psychiatric emergency. The HR decreased to 0.69 (95% CI: 0.44–0.99) when applying a less-restrictive grace period of 30-days, in contrast to 14 days, although the 95% CIs overlapped. When applying 30 days' grace period, fewer women would be categorized as discontinuing, i.e., they had to discontinue their medications for at least 30 days instead of 14 days to be defined as discontinuing. Although speculative, it is probable that women who get pregnant after discontinuing antidepressants 30 days may have planned their pregnancy even earlier than those who get pregnant 14 days after discontinuation. We have added this explanation into the discussion section (Line 347–351), but also acknowledge that any interpretation of these analyses should be made with caution.

19. Discussion: Please present and organize the Discussion as follows: a short, clear summary of the article's findings; what the study adds to existing research and where and why the results may differ from previous research; strengths and limitations of the study; implications and next steps for research, clinical practice, and/or public policy; one-paragraph conclusion.

**Response**: We have presented and organized the discussion as suggested.

20. Discussion: "No previous studies have investigated risk of psychiatric emergency in women who discontinued antidepressants before pregnancy." Please qualify this and any similar statements with "To the best of our knowledge…" or similar.

**Response**: We have added the statement as suggested.

21. Conclusions: "This large propensity score-matched cohort study suggests that discontinuing antidepressants specifically during pregnancy increases risk of psychiatric emergency…" Please avoid the use of language implying causality, here and throughout the manuscript.

**Response**: We now rephrased the sentences to avoid the use of language implying causality as suggested.

22. References: Please use the "Vancouver" style for reference formatting, and see our website for other reference guidelines https://journals.plos.org/plosmedicine/s/submission-guidelines#loc-references

**Response**: We have formatted the references based on the "Vancouver" style.

23. Table 2 and S5 Table: Please also provide the results of the unadjusted analyses.

**Response**: We have provided unadjusted hazard ratios as suggested. Please note that all the matched and well-balanced covariates were adjusted in the unadjusted hazard ratios due to the propensity score-matched design.

**Comments from the reviewers:**

**Reviewer #1, Peter Flom**:

I confine my remarks to statistical aspects of this paper. These were generally very well done, and I almost voted to accept, but I have a couple of suggestions that the authors might implement, especially if they have to make other revisions.

**Response**: We thank the reviewer for the positive comments about our study.

p. 2 Results - there was, in fact, some evidence of *lower* risk among those who discontinued before pregnancy.

**Response**: We agree with the reviewer that women who discontinued before pregnancy may have a "lower" risk than those who continued. As mentioned in the discussion section, women who discontinued antidepressants before pregnancy may represent a selected population with better disease control and pregnancy planning. To acknowledge the complexity of our presented results, we have also added more discussion on the reduced risk in the sensitivity analysis when applying 30 days' grace period to reflect this (L347–351).

p. 4 It would be good to give effect size estimates of the other studies.

The results of the meta-analysis are very odd. There must have been huge heterogeneity to get such a large confidence interval. The authors might discuss this.

**Response**: We agree with the reviewer that presenting the magnitude of the estimates will provide information on the heterogeneity of findings on this topic. We have added one sentence to describe the effect size estimates of the other studies. It reads, "*The magnitude of the relative risk of relapse for antidepressant discontinuation versus continuation ranges from 0.45 to 8.79."*

We have also rephrased our sentence to reflect this in the introduction section: *"However, all studies differed by the study populations, depression severity, time of discontinuation, relapse assessment, and a high degree of heterogeneity between studies was reported (I^2^=94%)."*

p, 7 Although the results of the propensity analysis were very good, they might be even better if the authors used splines of age as a continuous variable, rather than catgegorizing it.

**Response**: We thank the reviewer for this interesting suggestion. We have repeated our analyses by including age at pregnancy as linear splines with four knots and age at first affective disorders as linear splines with five knots at specified values based on age groups included in Table 1. The HR of psychiatric emergency among women who discontinued antidepressants before pregnancy was 0.81 (95% CI: 0.58–1.14) and during pregnancy 1.27 (95%CI: 1.01–1.59%), in comparison to women who continued antidepressants, which is similar to 0.84 (95% CI: 0.61–1.16) and 1.25 (95% CI: 1.00–1.55%) in the primary analysis. Therefore, we retain the primary analyses and report the new results as a sensitivity analysis (S6 Table in the supplementary materials).

**Reviewer #2:**

Overall, this is interesting, important, rigorous work. I appreciate the detailed description of the cohort and matching. Outcomes (emergency visit or admission) are somewhat narrow, as acknowledged by authors, in that this probably does not capture the spectrum of the burden of recurrent illness during pregnancy/postpartum and many (perhaps most) women are probably not making a decision to continue or discontinue medication during pregnancy based on those outcomes, unless they have previously experienced those events.

**Response**: We thank the reviewer for the positive comments about our study. We chose to focus on the most concerning and stressful outcome: psychiatric emergency or inpatient treatment since it is a proxy measure for the most severe exacerbation of symptoms/mental health crisis and may involve threats to safety, including self-harm and suicide [1] and thus impact the mother and her child significantly. In addition to psychiatric emergency and admissions, women with a mental health crisis can also present at psychiatric treatment facilities with suicide attempts. We also considered using suicide attempts as an alternative outcome. However, suicide attempts were rare, 0.4–0.5% in the matched cohorts, and therefore did not provide a meaningful outcome (Line 192–194).

We agree with the reviewer that we would not capture a complete spectrum of the burden of recurrent illness, in particular, mild to moderate episodes, which do not necessarily lead to psychiatric emergency room visits or admissions. We have included these considerations in our discussion section (Line 325–329). It reads,

"*Furthermore, our results should be interpreted with caution because a psychiatric emergency is a severe outcome. In contrast, a larger proportion of these women may have a recurrent episode that does not necessarily lead to emergency room visits or admissions, and future prospective clinical studies should also investigate less severe outcomes such as worsening of symptoms or affective instability."*

As the reviewer pointed out, the decision to discontinue or continue antidepressants may depend on previous disease history. To reflect this, we also examined whether the associations differed by previous psychiatric emergencies by defining patients with severe disorders as having admission, emergency room visits, or suicide attempts before the start of pregnancy and moderate disorders otherwise. As expected, the association between antidepressant discontinuation during pregnancy and risk of psychiatric emergency was stronger for women with severe disorders (HR=2.68, 95%CI: 0.91–7.89) than those with moderate disorders (HR=1.38, 95%CI: 1.00–1.89), although the confidence intervals overlapped (Line 278–284).

Several minor comments.

Abstract: It might be more accurate to say that there is limited evidence on continuing vs discontinuing antidepressants in pregnancy.

**Response**: We have rephrased the sentence in the abstract as suggested.

Introduction:

2-8% of pregnant women in Europe and 8-13% in the US receive an antidepressant prescription at some time during their pregnancy (versus continuous use).

Untreated/undertreated depression and anxiety should also be avoided to prevent maternal/fetal/infant death.

**Response**: We have revised the sentences to clarify that these percentages are not the prevalence of continuous users but antidepressant use at some point in their pregnancy.

We agree with the reviewer that untreated or undertreated depression and anxiety are associated with poor health outcomes in mothers and children, or at its worst, suicide or infanticide in the perinatal period. We have added this to the introduction section (Line 84–85).

Methods:

If 41.3% had a psychiatric diagnosis, what were the indications for antidepressant use in the rest of the cohort? This is brought up in methods and again the discussion. Would be helpful to understand if having a documented psychiatric diagnosis indicates a more severe illness.

**Response**: Most of the patients with affective disorders in Denmark are treated by general practitioners in Denmark[5]. The main indications for antidepressant treatment among pregnant women are depression and anxiety[6], as shown in our previous studies. We agree with the reviewers that those who received a psychiatric diagnosis may have a more severe illness. We have reflected on this in the discussion section (Discussion section, Line 372).

Can you comment on if there was a group of women who discontinued antidepressants before or during pregnancy and then restarted them during pregnancy or during postpartum?

**Response**: We agree with the reviewer that some women will restart their antidepressants during pregnancy or in the postpartum period. In our previous study, we found that one in three patients will restart antidepressant treatment within one year after antidepressant discontinuation in the general population [7]. The proportion restarting antidepressants may be lower among pregnant women due to the concerns of potential fetal risk. We speculate that women who discontinue antidepressants during pregnancy may restart their medication treatment, in particular, after childbirth [8] and thus reduce the risk of a psychiatric emergency. We have incorporated these considerations into the discussion (Line 317–323).

**Reviewer #3:**

The authors try to address the exceedingly important question of navigating the risk benefit decision with respect to antidepressant use during pregnancy: to continue or to discontinue AD treatment given the relative knowns and unknowns associated with fetal exposure to this class of medication. The analytic exercise performed is profoundly flawed and does not contribute to enhanced ability to navigate the clinical dilemma. Indeed, there are some conflicting data on the question of benefit of antidepressant continuation during pregnancy. And yet this paper looks at an outcome variable - psychiatric emergency /ER visit /psych admission with respect to AD discontinuation that does not inform in any way.

**Response**: We appreciate the comments made by Reviewer #3 and always welcome the opportunity to discuss our research and the inherent challenges in pharmacoepidemiological studies using administrative data. We believe that there is no single "ideal" study that can solve the clinical dilemma posed by the reviewer, but we do believe that our present study provides one of several steps towards assisting treatment decision-making in pregnancy and postpartum.

In terms of our chosen analyses, we have used state-of-the-art pharmacoepidemiological methods to approach the challenging need to make discontinuation and continuation groups comparable in terms of observed baseline covariates. In addition, we have taken into consideration, by design, the timing of antidepressant discontinuation. The generally positive comments from the other reviewers support our analytical strategy. It should be noted that these are methods used by some of the leading pharmacoepidemiological research groups, also using information from administrative data sources [9-12].

Do the authors make the argument that they have the ability to adjust for confounding based on the data in hand from an administrative database? The variables in the propensity score model are appropriate but the reader can have sparse confidence that data reflecting accurately those variables is obtainable. An even greater limitation of this analysis is the failure to know the status of subjects in each cohort at baseline: were these women euthymic at baseline, before they discontinued treatment? What value is the analysis if we do not know how women were clinically before looking at the outcome of interest? This may also be relevant as treatment of depression before, during and after pregnancy has certainly evolved across the interval of interest noted in the paper - 1996-2016.

**Response:** Our study is a cohort study based on administrative data. We agree with the reviewer that administrative data are not without limitations, as we have acknowledged this in our discussion (Page 17–18). It is correct that we have no information on whether the women were euthymic or not at baseline as no symptoms are recorded in our data. However, to account for this, in our propensity score matching, we took into consideration variables related to disease history before pregnancy and the severity of disorders measured in the 90 days prior to pregnancy, including co-medications and psychiatric admissions (Line 38–40, and 155–186). This was done to consider how the health status of the individual woman prior to pregnancy can influence our observed associations. When we categorized the propensity scores of antidepressant discontinuation into five groups in the unmatched cohort, we found that women with a high propensity score were more likely to discontinue their treatment (Figure). This suggests that the propensity score predicts antidepressant discontinuation well.

**Figure.** Proportion of antidepressant discontinuation during pregnancy among women who were on antidepressant treatment at the start of pregnancy (N=18,821)

Moreover, health administrative databases are a valuable source of data for studying the association between treatment and disease outcome. The use of administrative data to estimate the risks and benefits of medication use is common worldwide; these data are employed not only in the Nordic countries but also in the USA. One of the leading pharmacoepidemiological groups at the Harvard Medical School has published extensively in the scientific literature using Medicaid data [9-12] and provides convincing evidence on applying administrative data to conduct pharmacoepidemiological studies.

Based on national registers, we are able to include a representative study population covering women with mild to severe symptoms provides a valuable tool for pharmacoepidemiological research to evaluate the real-world effectiveness of medication use, which may be more generalizable than findings from studies including only patients with severe symptoms. Furthermore, administrative data are helpful in finding a sizable study population with rare exposures or health outcomes, such as severe exacerbation of symptoms/mental health crisis in our study. The linkage between registers also allows adjustment for a wide range of confounders such as demographics and clinical treatment, and proxies of disease severity. We agree with the reviewer that treatment of depression before, during, and after pregnancy has evolved over time. For this reason, we have included calendar time as a variable in the matching (Line 177–178). In summary, with our different approaches, we attempted to provide the most unbiased estimates of the associations as is possible. Our interpretation of the results is made bearing in mind the limitations of the utilized data sources, and we discuss the clinical significance of our observed effect sizes.

Two other smaller issues: First, the reference to adverse effects of AD exposure during pregnancy being associated with persistent pulmonary hypertension of the newborn is puzzling as the concern has been put to rest in multiple reports. And yet these authors note as an outcome of concern with respect to fetal exposure to AD. Second, there is no discussion as to why there is no observation of increased frequency of the outcome postpartum which would be expected.

**Response:** We agree with the reviewer that the association between antidepressant exposure during pregnancy and persistent pulmonary hypertension in neonates (PPHN) has been studied extensively but do not agree that it has been put to rest as it is still being discussed and is a concerning outcome. A review paper published in 2019 performed a comprehensive analysis of 11 published studies and reported an increased risk of PPHN for newborns whose mothers used antidepressants during pregnancy [13]. Therefore, PPHN is still a concern, although it is an infrequent complication.

We have expanded our discussion as to why there is no increased risk of the outcome in the postpartum (HR=1.15, 95% CI: 0.85–1.54). We have added our interpretation regarding no increased risk of psychiatric emergency in the postpartum period following antidepressant discontinuation during pregnancy as suggested. Briefly, we speculate that women who discontinue antidepressants during pregnancy may restart their medication treatment, in particular, after childbirth and thus reduce the risk of a psychiatric emergency. However, the 95% CIs overlapped, and the difference in the risk noted should be interpreted with caution (Line 317–323).

There is a responsibility as studies get conducted in this area to make an effort to refine the risk benefit decision regarding the use of AD before, during and after pregnancy in reproductive age women. This manuscript does not advance the effort.

**Response**: We appreciate the comments from Reviewer #3, but we do believe that our manuscript informs clinicians and patients of the risk of severe relapse, which is important but should be considered in light of other potential worrying outcomes in this population (Line 325–329). Treatment decisions related to depression during pregnancy are complicated and are made mainly based on the clinical characteristics of each individual woman, the uncertainty and magnitude of the risks, and the woman's personal preferences, together with advice from her treating physicians. Most women prefer an active role in treatment decisions, which requires evidence-based information, including data on women's perspectives [14]. However, evidence on women's perspectives is sparse and inconsistent. We believe there is no single study that can enhance the risk-benefit decisions. In contrast, multiple high-quality studies with different study populations and methodological triangulation are warranted to provide sufficient evidence, so we argue that our study can add to the joint evidence base in this field.

**Reviewer #4:**

This manuscript is a cohort study that examines the effects of antidepressant discontinuation before and during pregnancy. The authors report an increased risk of psychiatric emergency in women who discontinue antidepressants during pregnancy. The risk is smaller than has been previously observed in the literature but clinically significant. There are several strengths of this manuscript including large sample size, transparent and thoughtful methodology, use of propensity score matching and a well-written paper. There are some minor critiques that I have of this manuscript that could improve it with revision but overall it is an important and meaningful work.

**Response**: We thank the reviewer for the positive comments about our study.

(1) Please define which medications are defined as antidepressants in the methods section. It is clear this is SSRI, SNRI, TCA and MAOI from table 1 but this should be mentioned in the text of the manuscript.

**Response**: We have moved the description of classes of antidepressants to exposure of interest in the methods section (Methods section, Line 121–124).

(2) I wish the authors discussed more extensively the evidence (or lack thereof) of congenital malformations with antidepressant exposure. This is important when discussing the risk/benefits of antidepressant discontinuation during pregnancy.

**Response**: We have added a few sentences on the evidence of congenital malformations with antidepressant exposure (Line 362–364). Briefly, although the available data suggest that antidepressants are not major teratogens [15], the evidence is inconclusive for longer-term outcomes in offspring [16].

(3) Although the absolute risk of psychiatric emergency is smaller than previous studies, I am not sure I would necessarily qualify it as that small of an absolute risk (not to mention it is not a small relative risk). The NNT seems to be around 77 and is probably much higher in individuals with moderate-to-severe illness or at the higher end of your propensity score.

**Response**: We thank the reviewer for pointing this out. We have removed the statement from the abstract and author summary.

(4) I also wish the authors commented on the high rate of women that discontinued antidepressant medication during pregnancy. It would be useful to examine whether there was any relationship between propensity score and decision to discontinue medication during pregnancy.

**Response**: We thank the reviewer for the great suggestion. As shown in S3 Figure, women who discontinue during pregnancy had a higher propensity score than women who continued, although the confidence intervals largely overlapped. We also categorized propensity scores into five groups based on the distribution and found that a higher propensity score was associated with an increased odds of discontinuing antidepressant treatment, as illustrated in Figure below.

**Figure.** Proportion of antidepressant discontinuation during pregnancy among women who were on antidepressant treatment at the start of pregnancy (N=18,821)

**References**

1.	Appleby L, Mortensen PB, Faragher EB. Suicide and other causes of mortality after postpartum psychiatric admission. *Br J Psychiatry*. 1998;173:209-11. PMID: 9926095.

2.	Grigoriadis S, Wilton AS, Kurdyak PA, Rhodes AE, VonderPorten EH, Levitt A, et al. Perinatal suicide in Ontario, Canada: a 15-year population-based study. *CMAJ*, 2017;189(34):E1085-e92. PMID: 28847780.

3.	Swanson SA, Hernandez-Diaz S, Palmsten K, Mogun H, Olfson M, Huybrechts KF. Methodological considerations in assessing the effectiveness of antidepressant medication continuation during pregnancy using administrative data. *Pharmacoepidemiol Drug Saf.* 2015;24(9):934-42. Epub 2015/06/06. doi: 10.1002/pds.3798. PMID: 26045370.

4.	Bushnell GA, Sturmer T, White A, Pate V, Swanson SA, Azrael D, et al. Predicting persistence to antidepressant treatment in administrative claims data: Considering the influence of refill delays and prior persistence on other medications. *J Affect Disord*. 2016;196:138-47. Epub 2016/02/28. doi: 10.1016/j.jad.2016.02.012. PMID: 26921866.

5.	Musliner KL, Liu X, Gasse C, Christensen KS, Wimberley T, Munk-Olsen T. Incidence of medically treated depression in Denmark among individuals 15-44 years old: a comprehensive overview based on population registers. *Acta psychiatr Scand*. 2019;139(6):548-57. Epub 2019/03/26. doi: 10.1111/acps.13028. PMID: 30908590.

6.	Liu X, Musliner KL, Agerbo E, Ingstrup KG, Sun Y, Yu Y, et al. Treatment indications for antidepressants prescribed to pregnant women: A population-based descriptive study from Denmark. *Pharmacoepidemiol Drug Saf.* 2020;29(3):347-51.

7.	Liu X, Momen NC, Molenaar N, Rommel AS, Bergink V, Munk-Olsen T. Discontinuation of antidepressants: Is there a minimum time on treatment that will reduce relapse risk? Journal of affective disorders. 2021;290:254-60. Epub 2021/05/20. doi: 10.1016/j.jad.2021.04.045. PMID: 34010750.

8.	Trinh NT, Nordeng HM, Bandoli G, Eberhard-Gran M, Lupattelli A. Antidepressant and mental health care utilization in pregnant women with depression and/or anxiety: an interrupted time-series analysis. *medRxiv.* 2021:2021.09.29.21264292. doi: 10.1101/2021.09.29.21264292.

9.	Patorno E, Huybrechts KF, Bateman BT, Cohen JM, Desai RJ, Mogun H, et al. Lithium Use in Pregnancy and the Risk of Cardiac Malformations. *N Engl J Med*. 2017;376(23):2245-54. Epub 2017/06/08. doi: 10.1056/NEJMoa1612222. PMID: 28591541.

10.	Huybrechts KF, Palmsten K, Avorn J, Cohen LS, Holmes LB, Franklin JM, et al. Antidepressant use in pregnancy and the risk of cardiac defects. *N Engl J Med*. 2014;370(25):2397-407. Epub 2014/06/19. doi: 10.1056/NEJMoa1312828. PMID: 24941178.

11.	Huybrechts KF, Hernández-Díaz S, Patorno E, Desai RJ, Mogun H, Dejene SZ, et al. Antipsychotic Use in Pregnancy and the Risk for Congenital Malformations. *JAMA psychiatry*. 2016;73(9):938-46. Epub 2016/08/20. doi: 10.1001/jamapsychiatry.2016.1520. PMID: 27540849.

12.	Huybrechts KF, Hernández-Díaz S, Straub L, Gray KJ, Zhu Y, Patorno E, et al. Association of Maternal First-Trimester Ondansetron Use With Cardiac Malformations and Oral Clefts in Offspring. *JAMA*. 2018;320(23):2429-37. Epub 2018/12/19. doi: 10.1001/jama.2018.18307. PMID: 30561479.

13.	Masarwa R, Bar-Oz B, Gorelik E, Reif S, Perlman A, Matok I. Prenatal exposure to selective serotonin reuptake inhibitors and serotonin norepinephrine reuptake inhibitors and risk for persistent pulmonary hypertension of the newborn: a systematic review, meta-analysis, and network meta-analysis. *AJOG*. 2019;220(1):57.e1-.e13. Epub 2018/09/01. doi: 10.1016/j.ajog.2018.08.030. d PMID: 30170040.

14.	Patel SR, Wisner KL. Decision making for depression treatment during pregnancy and the postpartum period. *Depression and anxiety*. 2011;28(7):589-95. Epub 2011/06/18. doi: 10.1002/da.20844. PMID: 21681871.

15.	Gao SY, Wu QJ, Sun C, Zhang TN, Shen ZQ, Liu CX, et al. Selective serotonin reuptake inhibitor use during early pregnancy and congenital malformations: a systematic review and meta-analysis of cohort studies of more than 9 million births. *BMC Med*. 2018;16(1):205. Epub 2018/11/13. doi: 10.1186/s12916-018-1193-5. PMID: 30415641.

16.	Rommel AS, Bergink V, Liu X, Munk-Olsen T, Molenaar NM. Long-Term Effects of Intrauterine Exposure to Antidepressants on Physical, Neurodevelopmental, and Psychiatric Outcomes: A Systematic Review. *J Clin Psychiat*. 2020;81(3). Epub 2020/05/16. doi: 10.4088/JCP.19r12965. PMID: 32412703.
