# Supplementary material for: Antidepressant discontinuation before or during pregnancy and risk of psychiatric emergency in Denmark: A population-based propensity score–matched cohort study
Source: PLoS Med. 2022 Jan 31;19(1):e1003895. doi: 10.1371/journal.pmed.1003895 (PMC8843130; doi:10.1371/journal.pmed.1003895)
Supplement: Attachment — Submitted filename: Rebuttal letter_3rd.docx [file pmed.1003895.s015.docx]

Dear Dr. Caitlin Moyer,

Thank you for sending us the comments and suggestions from the editors. Please find below a detailed point-by-point response to all comments (editors' comments in black, our replies in blue). The line numbers mentioned are based on the revised manuscript with track changes.

**Requests from Editors:**

1. Title: Please capitalize the first word of the subtitle: "Antidepressant discontinuation before or during pregnancy and risk of psychiatric emergency: A population-based propensity score-matched cohort study" and please update this in the manuscript submission system.

**Response:** We have changed the title as suggested.

2. Data availability statement: Thank you for providing a statement regarding the availability of the data underlying your study. Please add the weblinks and contact email address(es) for the registers used in the study (e.g. <https://econ.au.dk/the-national-centre-for-register-based-research/danish-registers/the-danish-civil-registration-system-cpr>; <https://econ.au.dk/the-national-centre-for-register-based-research/danish-registers/the-national-patient-register>; <https://econ.au.dk/the-national-centre-for-register-based-research/danish-registers/the-danish-psychiatric-central-research-register>; <https://econ.au.dk/the-national-centre-for-register-based-research/danish-registers/the-medical-register-of-births-and-deaths> if these links are applicable. Please also provide a link and contact information for the Danish National Prescription Registry).

**Response:** We have provided the web links for the registers (<https://econ.au.dk/the-national-centre-for-register-based-research/danish-registers> and <https://kea.au.dk/research/registers-and-biobank/the-danish-national-health-service-prescription-database>) and the contact email address (cbp@econ.au.dk).

3. Abstract: Methods: Line 34: Please revise to "We carried out a propensity score-matched cohort study of women with singleton live births between…" or similar. Please also note the setting of the study (Denmark) in the first sentence.

**Response:** We have rephrased the sentence as suggested (Line 34–35).

4. Abstract: Methods: Line 35-36: Please broadly define the categories of "antidepressants" considered.

**Response:** We have rephrased the sentence to *"… who redeemed an antidepressant prescription in the 90 days before the pregnancy, identified with Anatomical Therapeutic Chemical code N06A*" to define antidepressants broadly.

5. Abstract: Methods and Findings: Please avoid the use of italics for emphasis.

**Response:** We have formatted the abstract, removing italics throughout the text.

6. Abstract: Methods and Findings: Please also provide the cumulative incidence for those who discontinued before pregnancy compared to those who did not.

**Response:** We have added the cumulative incidence to the abstract as suggested (Line 46–48).

7. Abstract: Conclusions: Please revise to: "In this study, we found that discontinuing antidepressant medication during pregnancy (but not before) is associated with an apparent increased risk of psychiatric emergency compared to continuing treatment across pregnancy."

**Response:** We have revised the conclusion in the abstract as suggested.

8. Author summary: What do these findings mean? We suggest revising to: "Our findings add to the limited evidence on the relationship between continued antidepressant treatment during pregnancy and perinatal psychiatric emergency."

**Response:** We have revised the author summary as suggested.

9. Introduction: Line 97: Please avoid the use of italics for emphasis here and throughout the text.

**Response:** We have removed the italics for emphasis throughout the text as suggested.

10. Methods: Line 105-106: Thank you for your response indicating that there was no prospectively developed protocol for the study. Please include a sentence noting this in the main text, early on in the Methods section, similar to: "There is no documented analysis plan associated with the study. We planned our analyses on a detailed discussion among the authors and agreed on an outline for how the work would be done, as described in the Methods. Changes to the pre-planned analyses include…" and please specifically note the changes to the analyses that were implemented post-hoc, e.g. in response to peer reviewer comments. We ask that you please indicate in the text: (1) the specific hypotheses you intended to test, (2) the analytical methods by which you planned to test them, (3) the analyses you actually performed, and (4) when reported analyses differ from those that were planned, transparent explanations for differences that affect the reliability of the study's results.

If a reported analysis was performed based on an interesting but unanticipated pattern in the data, please be clear that the analysis was data-driven.

**Response:** We have added sentences to indicate the change to the pre-planned analyses (Line 107–112). We have also indicated in the text to justify the change of the analysis plan (L178–181, 299–301, and S6 Table).

11. Methods: Line 110-111: Thank you for including the detailed information in the Supporting Information files. In the main text, please do at least mention the names of the registers (e.g. the register from which pregnancies and births were identified, etc).

**Response:** We have incorporated the registers that were applied to identify the study population (Line 117–122).

12. Methods: Line 160: Please briefly explain "mandatory school" in terms of what this indicates.

**Response:** The mandatory school comprises nine school years (grades 1–9) in Denmark. We have added this information to the methods section (L166).

13. Methods: Line 165-166: Please either list the 19 comorbid conditions here, or provide a supporting information file with the list of conditions.

**Response:** We have provided supporting information in the S2 Charlson comorbidity index in the supplementary material.

14. Methods: Line 216: Please list the imbalance covariates adjusted for in the adjusted analyses.

**Response:** We have included the imbalance covariates adjusted for in the adjusted analyses (Line 244–249).

15. Results: Line 249-250: Please add "significantly" to the sentence: "Antidepressant discontinuation before pregnancy was not significantly associated with…"

**Response:** We have added "significantly" to the sentence as suggested.

16. Results: Line 251-253: Please provide the results (for the interaction) in the text that support this statement: "The risk of psychiatric emergency associated with antidepressant discontinuation before pregnancy did not differ between the pregnancy and the postpartum periods."

**Response:** We have provided the p-value for the interaction in the results to support the statement (Line 261).

17. Results: Line 257: Please clarify to "A total of 202 women who discontinued during pregnancy…" or similar.

**Response:** We have rephrased the sentences for clarification (Line 253 and 265).

18. Results: Line 263: Please clarify to "The risk of psychiatric emergency associated with antidepressant discontinuation during pregnancy…" if accurate.

**Response:** We have rephrased the sentence for clarification (Line 272). Now it reads, " *The risk of psychiatric emergency associated with antidepressant discontinuation was increased during the observation time of pregnancy (HR=1.52, 95%CI: 1.08–2.15, p=0.016) but not during the postpartum period (HR=1.09, 95%CI: 0.80–1.47, p=0.596)".*

19. Results: Line 265: We suggest revising to: "There was no statistically significant interaction between antidepressant discontinuation during pregnancy and time of observation of psychiatric emergency (p=0.153)." or similar.

**Response:** We have reworded the sentence as the editor suggests (Line 273–274).

20. Results: Lines 277-279: We suggest revising to "The associations remained similar, though they did not reach statistical significance: the HR was 0.74 (95%CI: 0.47–1.17, p=0.137) for SSRI discontinuation before pregnancy and 1.16 (95%CI: 0.88–1.53, p=0.302) for SSRI discontinuation during pregnancy, compared to women who continued SSRIs." or similar.

**Response:** We have revised the sentence as the editor suggests (Line 286–290).

21. Results: Line 281: Please clarify "having admission" in this sentence.

**Response:** We have reworded "having admission" as "being admitted to a psychiatric hospital" for clarification (Line 292).

22. Results: Line 282-285: We suggest revising to: "The association between antidepressant discontinuation during pregnancy and risk of psychiatric emergency was greater for women with severe disorders (HR=2.68, 95%CI: 0.91–7.89, p=0.708) than those with moderate disorders (HR=1.38, 95%CI: 1.00–1.89, p=0.050), although associations did not meet statistical significance and the confidence intervals overlapped."

**Response:** We have rephrased the sentences as suggested (Line 293–298).

23. Discussion: Lines 292-294: We suggest revising to: "Conducting a propensity score-matched cohort study, we found that antidepressant discontinuation during pregnancy was associated with an increased risk of psychiatric emergency during the perinatal period." as there was no significant interaction between psychiatric emergency during pregnancy and the 6 months following pregnancy.

**Response:** We have rephrased the sentences as suggested (Line 304–307).

24. Discussion: Line 300: Here and throughout, where multiple references are indicated within brackets, please do not include spaces [6,7,34].

**Response:** We have checked all the references to ensure that they all meet the formatting requirement.

25. Discussion: Lines 306-308: Please revise to "...the point estimate suggests that the increased risk of psychiatric emergencies following antidepressant discontinuation may be more pronounced among women with severe psychiatric disorders…"

**Response:** We have rephrased the sentence (Line 318–321).

26. Discussion: Lines 347-349: It would be helpful to further clarify this sentence, in terms of why the less-restrictive grace period would lead to a reversal of the direction of the association with the 14 day grace period. "It is probable that women who get pregnant after discontinuing antidepressants 30 days may have planned their pregnancy even earlier than those who get pregnant 14 days after discontinuation."

**Response:** We have rephrased the sentence for further clarification (Line 359–361). Now it reads, “*One explanation for this particular difference could relate to differences in pregnancy planning between the two groups; however, any explanations are at this point highly speculative, as we do not have information on reasons for starting and stopping medication use.*”

27. Discussion: Line 359: We suggest removing "(and will likely remain)" from the sentence.

**Response:** We have removed the phrase from the sentence (Line 373).

28. Discussion: Line 377: Please use "adherence" rather than "compliance" here and throughout the manuscript.

**Response:** We have changed compliance to adherence as suggested.

29. Discussion: Line 399-403: We suggest revising to: "However, we observed that the absolute risk difference of psychiatric emergency is low (cumulative incidence of 5.0% in women who discontinue versus 3.7% in women who continue antidepressants). While causality cannot be determined from this study, if the association we observed indicates a causal relationship, continuing antidepressant treatment across pregnancy may be effective in reducing psychiatric emergency risk."

**Response:** We have rephrased the sentences as suggested (Line 413–418).

30. Line 405: Please remove the "Competing Interests" section from the main text, and please ensure all information is complete and accurate in the Competing Interests section of the manuscript submission system.

**Response:** We have removed the competing interests from the main text.

31. Lines 405-426: Please remove the Funding, Contributors, and Data Sharing sections from the main text and please ensure all information is completely and accurately entered into the relevant sections of the manuscript submission system.

**Response:** We have removed the funding, contributors, and data sharing sections from the main text.

32. References: Please use the "Vancouver" style for reference formatting, and see our website for other reference guidelines <https://journals.plos.org/plosmedicine/s/submission-guidelines#loc-references>

Please avoid the use of italics.

**Response:** We have formatted the references and removed all the italics.

33. Figure 1: Please revise to "680,312 women aged 18 years or older" in the first box.

**Response:** Thank you for pointing this out. We have revised it accordingly.

34. Figure 2: For comparison, could the curves also be shown for those who discontinued antidepressants prior to pregnancy? Please provide the number at risk for each relevant time interval.

**Response:** We have provided the number at risk for each relevant time interval to the figures.

Women who discontinued antidepressants prior to pregnancy have distinct characteristics from women who discontinued antidepressants during pregnancy. Therefore, the cumulative incidence rates between these two groups may not be directly comparable. We have included Figure 2 (the original Figure 2 was changed to Figure 3) to illustrate the cumulative incidence of relapse among women who discontinued antidepressant treatment before pregnancy and their matched continuation group. The same y-scales were used for comparability.

35. Table 1: Please define all abbreviations used in the table. Please include a definition of "mandatory school" in the legend.

**Response:** We have defined all abbreviations used in Table 1 and included a definition of mandatory school in the legend.

36. Supporting Information file: Please submit a clean version of the file (without tracked changes). Please include both titles and legends for all figures and tables.

**Response:** We have included a clean version of the supporting file.

37. Supporting Information S2: Please provide a citation or other quantitative support for the sentence "The information is of very high quality."

**Response:** We have referred to reference 1 to support the statement of very high quality of the information in Supporting information S2.

38. S1 Figure: Please provide a descriptive legend for the figure, and please provide more information for the y-axis label.

**Response:** We have changed the y-axis label to "Probability density of women who discontinued antidepressants".

39. S4 Table: Please define all abbreviations used in the table. Please include a definition of "mandatory school" in the legend.

**Response:** We have defined all abbreviations used in S4 Table and included a definition of mandatory school in the legend.

40. STROBE Checklist: Please provide the checklist as a separate file.

**Response:** We have included STROBE Checklist as a separate file.

**Comments from Reviewers:**

Reviewer #1, Peter Flom: The authors have addressed my concerns and I now recommend publication.

Reviewer #2: I am satisfied with the revisions and have no additional comments to offer. The work remains important and although it is not possible to control for all confounders, etc, this does add to the field and could be used in helping clinicians/patients determine risk of continuing vs discontinuing medications preconception and during pregnancy.

Reviewer #4: The reviewers responded adequately to my critiques. I believe many of the important criticisms raised by other reviewers have also been adequately addressed in the limitations section and are well-acknowledged by the authors in the revised manuscript.

**Response:** We thank all the reviewers again for their inputs and comments.
